# Functional Genomic Identification of Predictors of Sensitivity and Mechanisms of Resistance to Multivalent Second-Generation TRAIL-R2 Agonists

**DOI:** 10.1158/1535-7163.MCT-21-0532

**Published:** 2022-01-27

**Authors:** Vera Grinkevitch, Mark Wappett, Nyree Crawford, Stacey Price, Andrea Lees, Christopher McCann, Katherine McAllister, Jochen Prehn, Jamie Young, Jess Bateson, Lewis Gallagher, Magali Michaut, Vivek Iyer, Aikaterini Chatzipli, Syd Barthorpe, Daniel Ciznadija, Ido Sloma, Amy Wesa, David A. Tice, Lodewyk Wessels, Mathew Garnett, Daniel B. Longley, Ultan McDermott, Simon S. McDade

**Affiliations:** 1 Wellcome Sanger Institute, Hinxton, Cambridge, United Kingdom.; 2 Patrick G. Johnston Centre for Cancer Research, Queen's University, Belfast, United Kingdom.; 3 Royal College of Surgeons Ireland, Dublin, Ireland.; 4 Division of Molecular Carcinogenesis, The Netherlands Cancer Institute, Amsterdam, the Netherlands.; 5 Champions Oncology Inc., Rockville, Maryland.; 6 Oncology R&D, AstraZeneca, Gaithersburg, Maryland.; 7 Delft Bioinformatics Lab, TU Delft, Delft, the Netherlands.

## Abstract

Multivalent second-generation TRAIL-R2 agonists are currently in late preclinical development and early clinical trials. Herein, we use a representative second-generation agent, MEDI3039, to address two major clinical challenges facing these agents: lack of predictive biomarkers to enable patient selection and emergence of resistance. Genome-wide CRISPR knockout screens were notable for the lack of resistance mechanisms beyond the canonical TRAIL-R2 pathway (caspase-8, FADD, BID) as well as p53 and BAX in TP53 wild-type models, whereas a CRISPR activatory screen identified cell death inhibitors MCL-1 and BCL-XL as mechanisms to suppress MEDI3039-induced cell death. High-throughput drug screening failed to identify genomic alterations associated with response to MEDI3039; however, transcriptomics analysis revealed striking association between MEDI3039 sensitivity and expression of core components of the extrinsic apoptotic pathway, most notably its main apoptotic effector caspase-8 in solid tumor cell lines. Further analyses of colorectal cell lines and patient-derived xenografts identified caspase-8 expression ratio to its endogenous regulator FLIP(L) as predictive of sensitivity to MEDI3039 in several major solid tumor types and a further subset indicated by caspase-8:MCL-1 ratio. Subsequent MEDI3039 combination screening of TRAIL-R2, caspase-8, FADD, and BID knockout models with 60 compounds with varying mechanisms of action identified two inhibitor of apoptosis proteins (IAP) that exhibited strong synergy with MEDI3039 that could reverse resistance only in BID-deleted models. In summary, we identify the ratios of caspase-8:FLIP(L) and caspase-8:MCL-1 as potential predictive biomarkers for second-generation TRAIL-R2 agonists and loss of key effectors such as FADD and caspase-8 as likely drivers of clinical resistance in solid tumors.

## Introduction

The cell surface receptor *TNFRSF10B* (DR5, TRAIL-R2) is commonly overexpressed on the surface of cancer cells, and in preclinical studies, its ligand TNF-related apoptosis-inducing ligand (TRAIL)-activated apoptosis selectively in cancer versus normal cells (1). Thus, TRAIL and its receptors were identified as promising targets for the selective killing of cancer cells. First-generation agonists (recombinant protein formulations and receptor-targeting agonistic antibodies) targeted either both TRAIL-R1 and -R2, for example, Dulanermin/AMG-95, or were receptor-specific, for example, mapatumamab/HGS-ETR1 (anti-TRAIL-R1) and contaumamab/AMG-655 (anti-TRAIL-R2; ref. 1). While some promising activity together with minimal toxicity was observed in early clinical trials, overall single-agent activity was limited (1). This restricted activity was due to several factors, most notably the inability of first-generation TRAIL-R–targeted therapeutics to trigger the level of receptor cross-linking required to efficiently trigger apoptosis. Insufficient drug exposure, high tumor expression of mediators of resistance and the lack of effective tumor biomarkers to predict for drug response also likely contributed to the relatively low levels of clinical activity. Several newer specific and more potent second-generation TRAIL-R2 agonists (e.g., GEN1029, INBRX-109) have been developed and are currently under evaluation in early-stage clinical trials (2, 3).

MEDI3039 is a novel highly potent TRAIL receptor agonist, specifically targeting TRAIL-R2 (DR5/*TNFRSF10B*; ref. 4). MEDI3039 is composed of tandem repeats of monomeric Tn3 subunits engineered to bind the extracellular domain of TRAIL-R2 with high affinity. The Tn3 subunit is a monobody protein scaffold based on the third fibronectin type III domain of tenascin C (4). Tn3 resembles an antibody variable region with 3 complementarity-determining regions (CDR)-like loops that can be randomized for selection of novel protein-binding affinities. From a phage-displayed Tn3 library, a Tn3 protein was isolated that specifically binds to TRAIL-R2 (4). Further optimization of binding affinity of the basic subunit was followed by testing of several multimeric forms, with six repeat units found to be optimal. The six TRAIL-R2–binding repeat units were then linked to polyethylene glycol-20 (PEG20) to improve *in vivo* half-life to generate MEDI3039, which has picomolar activity in a range of cancer cell line models and enhanced monotherapy activity *in vivo* compared with previously developed recombinant forms of TRAIL and first-generation agonists. MEDI3039 has previously been shown to induce cell death in multiple breast cancer cell lines and in the MDA-231T *in vivo* mammary fat pad and lung metastatic model. The sensitivity varied according to breast cancer molecular subgroup, with triple-negative breast cancer models notably being the most sensitive and estrogen receptor–positive models the least (5).

Two challenges exist for the effective clinical use of MEDI3039 and other multivalent second-generation TRAIL-R2 agonists: first, the lack of robust biomarkers to identify those tumors most likely to respond to treatment; and second, the high likelihood of resistance emerging as with all highly selective receptor-targeted therapies to date (6). In this study, we sought to address these important issues using pharmacogenomic and CRISPR screens in a range of cancer types.

## Materials and Methods

### Materials

All cell culture was performed in either RPMI or DMEM/F12 medium (according to the manufacturer's recommendations) and supplemented with 5% FBS and penicillin/streptomycin. Cells were maintained at 37°C and 5% CO_2_ during culture. Cell lines were acquired from the ATCC (MSTO-211H, SUP-T1, NCI-H28, NCI-H2804, and HCT-116) or RIKEN (PC-9) cancer cell line repositories. All cell lines were tested for *Mycoplasma* prior to experiments using the MycoAlert Mycoplasma Detection Kit (Lonza). The identity of all cell lines used in this article was confirmed using a panel of 96 single-nucleotide polymorphisms (SNP) used previously for cell line authentication (Sequenom).

### Western blot analysis

Standard Western blotting was carried as described previously (7). PARP, caspase-8, caspase-3, TRAIL-R2, and Bid antibodies were purchased from Cell Signaling Technology, cIAP1 from Enzo, FADD from BD Biosciences, FLIP antibody from AdipoGen, and β-actin from Sigma. For absolute quantification of FLIP and caspase-8, images were captured using an Odyssey Imaging System (LICOR) at 12-bit dynamic range. Quantification of protein expression amounts was conducted as described previously using recombinant proteins and a HeLa cell line standard (8).

### Annexin V/propidium iodide staining

Propidium iodide (PI; Sigma), FITC-tagged AnnexinV (BD Biosciences), and Hoescht stain (Invitrogen) were utilized to quantify cell death through high content microscopy on an Array Scan XTI microscope (Thermo Scientific).

### Drug sensitivity screen in a panel of cancer cell lines

Cells were seeded in 384-well microplates at approximately 15% confluency in culture medium with 10% FBS and penicillin/streptomycin. The optimal cell number for each cell line was determined to ensure that each was in growth phase at the end of the assay (∼85% confluency). The viability of 758 cancer cell lines was assayed following treatment for 6 days with a concentration range of the TRAIL agonist MEDI3039 (maximum concentration 5 nmol/L, 7 concentration points and 1,000-fold range; Supplementary Table S9). Viability was assessed using CellTiterGlo and IC_50_/AUC values calculated as previously published (9).

### Statistical models of drug response

An ANOVA model was fitted to correlate MEDI3039 response with the status of Cancer Functional Events (CFEs), as described in ref. 9. A drug–response vector consisting of 758 IC_50_ values from treatment of the cell lines was constructed for MEDI3039. The model was linear (no interaction terms) with dependent variables represented by the described vector and factors including tissue type, microsatellite instability status (for the cancer types with positive samples for this feature) and the status of a CFE (one model for each CFE). These CFEs consisted of driver mutations in any of 270 cancer genes and copy number gains/losses in 470 genomic regions found to be recurrent in human cancers.

### Gene expression data

Microarray data for the cancer cell line panel was generated using the Human Genome U219 96-Array (Affymetrix) and as described previously (9). It was analyzed on the Human Genome U219 96-Array Plate using the Gene Titan MC instrument (Affymetrix). The robust multi-array analysis (RMA) algorithm was used to establish intensity values for each of 18,562 loci. The data was normalized using RMA and analyzed at the probeset level (10). Raw data was deposited in ArrayExpress (accession number: E-MTAB-3610). The RMA processed dataset is available at http://www.cancerrxgene.org/gdsc1000/. Microarray data for the colorectal PDX models was also generated using the Human Genome U219 array and processed as above.

MEDI3039 AUC values were split into two datasets based on solid and hematologic cell line identity. Each dataset was partitioned into sensitive/resistant bins based on the bimodal mid-point of the density distribution of the IC_50_ values (11, 12). The identity of the cell lines in the sensitive and resistant bins was used to partition the matching mRNA microarray expression dataset (9). These data were generated on the Affymetrix HG U219 platform, normalized using RMA and analyzed at the probeset level (10). Differential expression analysis was performed using an unpaired two-group limma analysis in R and probesets were mapped to gene symbols using core Bioconductor annotation (13). These probesets were fed into the VSURF random forest package (14). vSURF performs variable selection in a stepwise manner that implements backward elimination of variables and then forward selection, adding variables (probesets) that collectively reduce the predictive error rate (out of box error).

The ability of the 9 probeset signature to predict the bimodal category of sensitivity was tested using the R package ROCR. The average expression of the 9 probesets for each cell line was calculated and the expression for each probeset was weighted according to the negative log_10_-adjusted *P* value of the bimodal low versus bimodal high ANOVA analysis using the following formula:




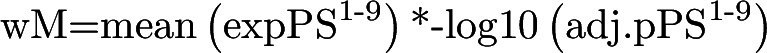




where wM = weighted mean, expPS is probeset expression, and adj.pPS is probeset adjusted *P* value bimodal high versus bimodal low.

### Genome-wide CRISPR/Cas9 library transduction for drug resistance gene detection

Generation of Cas9-expressing cancer cell lines and functional assessment. The cancer cell lines were transduced with a lentivirus produced from the Cas9 expression vector (Addgene, #68343). Blasticidin selection was initiated 2 days after transduction at 50 μg/mL for both cell lines. To assess the ability of Cas9-expressing cells to efficiently silence full-length gene expression, cells were transduced with a lentivirus produced from the Cas9 reporter vector – the pKLV2-U6gRNA5(gGFP)-PGKBFP2AGFP-W vector. This vector contains both a GFP-expressing cassette as well as a gRNA targeting GFP-efficient Cas9 activity would therefore be expected to result in silencing of GFP signal. The ratio of BFP only and GFP–BFP–double positive cells were analyzed on a BD LSRFortessa instrument (BD Biosciences) 3 days posttransduction for cancer cells. The data were subsequently analyzed using FlowJo. Transduced Cas9 cells showed high BFP expression but loss of GFP signal, indicating that the majority of cells express active Cas9.

#### Generation of genome-wide CRISPR/Cas9 libraries and positive selection screens

Libraries were constructed as described before with a minor modification (15). pKLV2-U6gRNA5(*Bbs*I)-PGKpuro2ABFP-W was used (Addgene, #50946). A total of 6 × 10^7^ cells were infected with a predetermined volume of the genome-wide gRNA lentiviral supernatant to ensure transduction MOI of 0.3, at which most cells receive only one genetic perturbation and therefore the gRNA library has a coverage of >200 cells expressing each gRNA. Each cell line was transduced in duplicate. Two days after transduction, the cells were selected with puromycin at 2–3 μg/mL for 5–6 days and further cultured. After puromycin selection, cells were maintained in culture for 14 days to allow for complete depletion of protein products of targeted genes, following which cells were split into equal numbers as duplicates for vehicle (no treatment) versus recombinant TRAIL (rTRAIL) treatment (IC_60–70_ values for each cell line - MSTO-211H cells were treated with 40 ng/mL rTRAIL on day 1 and 65 ng/mL day 3; PC-9 cells were treated with 50 ng/mL rTRAIL on days 1 and 4). After 10 days, cells from each of the duplicate vehicle and rTRAIL experimental arms were harvested and submitted separately for PCR and Illumina sequencing. The vehicle cells were used as a control for genes that when silenced increase the proliferation rate of cells and therefore would contribute disproportionately to gRNA enrichment in the rTRAIL-treated cells.

#### Illumina sequencing of gRNAs and statistical analysis

Genomic DNA extraction and Illumina sequencing of gRNAs were conducted as described previously (15). In brief, 72 μg of total extracted DNA was used to set up 36 PCR reactions (2 μg each) using 10 μmol/L concentrations of forward and reverse primers following which PCR products were purified using spin columns before a second PCR reaction was carried out to incorporate indexing primers for each sample. DNA was purified using SPRI beads and submitted for Illumina sequencing.

F primer: ACACTCTTTCCCTACACGACGCTCTTCCGATCT CTTGTGGAAAGGACGAAACA
R primer: TCGGCATTCCTGCTGAACCGCTCTTCCGATCTCTAAAGCGCATGCTCCAGA

Enrichment and depletion of guides and genes were analyzed using the MAGeCK statistical package (ver 0.5.4) by comparing read counts from each cell line with counts from matching DMSO cells, after comparing each to counts from the plasmid gRNA library (https://sourceforge.net/projects/mageck/; ref. 16). As an initial quality control assessment, we confirmed by gene-set enrichment analysis that those sgRNA most significantly depleted in the DMSO-treated cells compared with baseline plasmid representation were those targeting core essential biological processes for cell survival. We confirmed that the most significantly depleted pathways contained genes from the RNA polymerase, ribosome, proteasome, and spliceosome families, known core essential genes.

### Genome-wide CRISPR activation screens

#### Generation of MS2-p65-HSF1 and dCas9-VP64 stably expressing cancer cells

The human CRISPR SAM library consists of three components: a nucleolytically inactive Cas9-VP64 fusion, a gRNA incorporating two MS2 RNA aptamers at the tetraloop and stem-loop 2 and the MS2-P65-HSF1 plasmid that expresses the activation helper protein. We initially transduced 293T cells with either the lenti MS2-P65-HSF1_Hygro or the lenti dCAS9-VP64_Blast plasmids together with the packaging plasmid psPAX2 and envelope plasmid pMD2.G to generate virus (17). We then transduced the cancer cells with each lentivirus sequentially, selecting for cells with integration of virus using hygromycin and blasticidin. Cells stably resistant for both antibiotics were then used for transduction with the gRNA library.

#### Generation of genome-wide CRISPR activation libraries and positive selection screens

To generate the pooled lentiviral gRNA library of 70,297 guides targeting 18,965 coding genes, the lenti sgRNA(MS2)_puro pooled library was used to generate lentivirus from 293T cells together with psPax2 and pMD2.G (Addgene #12260, #12259) packaging and envelope plasmids. The gRNA library lentivirus was then used to transduce the cancer cell line stably expressing integrated for the lenti MS2-P65-HSF1_Hygro and the lenti dCAS9-VP64_Blast plasmids as described above. We transduced the cancer cells at MOI < 0.3 to ensure that most cells receive only one genetic perturbation. Cells were selected in puromycin for 10 days prior to the start of the drug resistance screen. Following selection, cells were maintained in culture for 14 days to allow for complete depletion of protein products of targeted genes, following which cells were treated with either DMSO (control) or rTRAIL treatment (drug replaced twice per week). After 10 days of rTRAIL drug selection, cells from each of the duplicate DMSO and rTRAIL experimental arms were harvested and submitted separately for PCR and Illumina sequencing. The lenti MS2-P65-HSF1_Hygro, lenti CAS9-VP64_Blast and lenti sgRNA(MS2)_puro plasmids were gifts from Feng Zhang (Addgene plasmid # 61426, #61425, #1000000074).

#### Illumina sequencing of gRNAs and statistical analysis

Genomic DNA extraction and Illumina sequencing of gRNAs were conducted as described previously (15). Enrichment and depletion of guides and genes were analyzed using the MAGeCK (version 0.5.8) statistical package by comparing read counts from each cell line with counts from matching DMSO cells, after comparing each to counts from the plasmid gRNA library (ref. 16).

#### Creation of isogenic cell lines with deletions in the apoptotic pathway

To create isogenic cell lines deleted for the gene of interest we used the Dharmacon Edit-R predesigned CRISPR RNA (crRNA) system (https://horizondiscovery.com/en/brands/edit-r; Supplementary Table S16). On day 0 before transfection, the cells were plated on a 96-well plate. The next day (cells at 40% confluency), the medium was replaced with 100 μL of medium and synthetic Dharmacon crRNA. The crRNA was reconstituted by mixing 5 nmol of crRNA with 5 nmol of tracrRNA in 20 μL volume of OPTIMEM medium, following which 0.5 μL of DharmaFECT Duo was diluted in 24.5 μL of OPTIMEM and added into the mixture. The final volume was then incubated for 20 minutes at room temperature. On day 2 posttransfection, the cells were transferred into a 12-well plate, and on day 7 the cells were selected using recombinant TRAIL for 1 week.

#### Combinatorial drug screens to resensitize MEDI3039-resistant isogenic cells

Isogenic cells expressing Cas9 and engineered using synthetic gRNA to silence the apoptotic pathways genes *FADD*, *CASP8*, *BID*, and *TNFRSF10B* (*DR5*) were derived from MSTO-211H, NCI-H28, and H2804 mesothelioma cells. They were screened together with their matching parental cell line against a panel of 60 compounds (including MEDI3039) over 6 days, and where each compound was screened over a 7-point concentration range and 1,000-fold difference from lowest to highest concentration (Supplementary Table S8). In addition, the panel of 60 compounds was screened against these cell lines combined with a fixed concentration of MEDI3039 (equivalent to IC_90_ values of the parental cell lines -100 pmol/L; Supplementary Table S15). Viability was assessed using CellTiterGlo and IC_50_/AUC values calculated as previously published for the single agent data (9). For the drug combination data, we calculated ΔAUC values for each drug/MEDI3039 combination.

#### Calculation of AUC and ΔAUC values from cell line viability data

We derived the AUC parameter from the 6-day cell line viability data to identify cell lines that are sensitive to a specific compound, with decreasing AUC associated with increasing sensitivity. The AUCs were computed using a trapezoid integration below the measured viability at each of the 7 concentrations of the dose–response curve and scaled so that a constant viability of 1 gives AUC of 1. We derived a parameter, the ΔAUC, to best capture those examples of two-drug combinations with rTRAIL that could be classified as synergistic for the purposes of this study. For two drugs, each at a given concentration, we used the Bliss model to compute the expected viability of the cell line when exposed to the drug pair (product of the viability at each single drug concentration; ref. 18). This defines the expected dose–response curve on the 7 measured concentrations of the library drug used in combination with rTRAIL. The ΔAUC is defined as the difference between the AUC below the expected dose–response curve and the AUC below the observed dose–response curve as experimentally measured in the presence of the two drugs.

### Data and materials availability

All data are available in Supplementary tables or through links indicated in methods above.

## Results

### CRISPR/Cas9 screens to systematically uncover resistance pathways to TRAIL-mediated apoptosis

**Figure 1.**
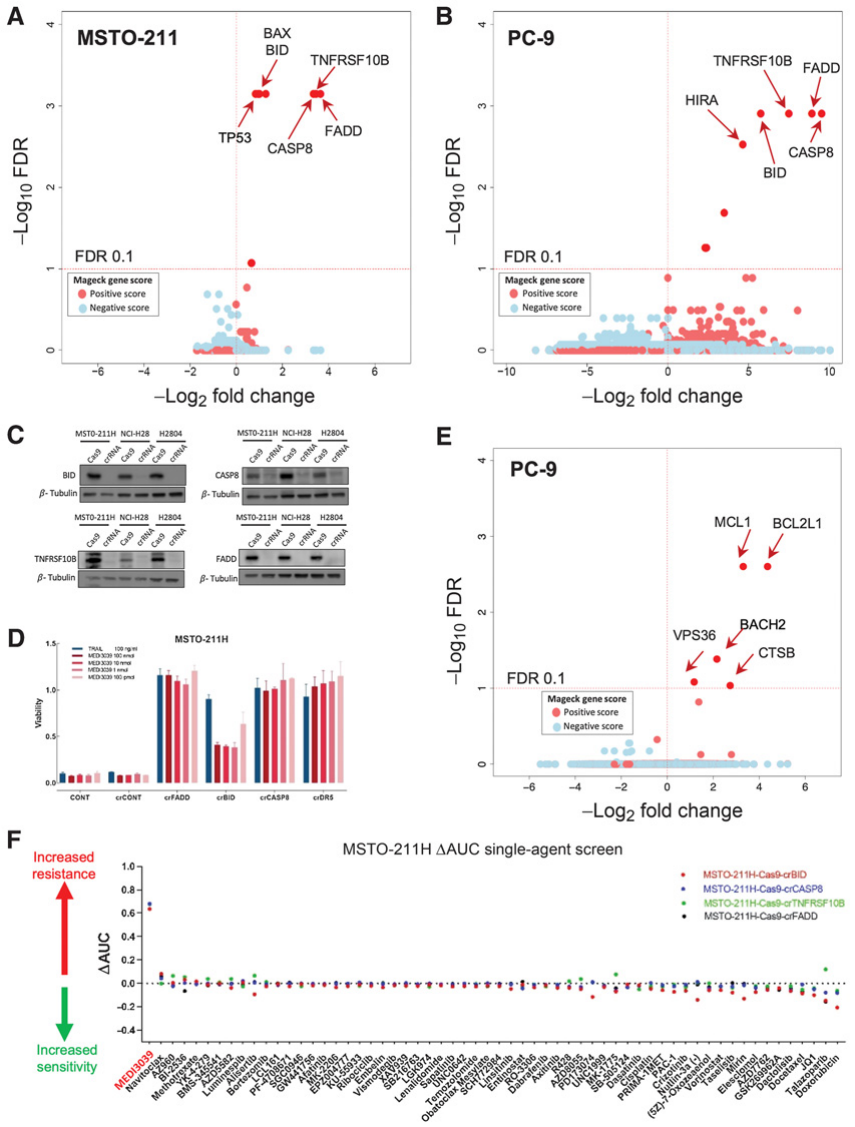
Silencing of apoptotic pathway genes confers resistance to agonists of the death receptor pathway. Volcano plots of genes enriched or depleted for gRNA when rTRAIL-treated cells were compared with DMSO vehicle control cells following transduction with a genome-wide CRISPR/Cas9 library in MSTO-211H mesothelioma (**A**) and PC-9 lung adenocarcinoma (**B**) cells. Each dot represents gene-wise scores for MAGeCK gene level analysis with genes of interest highlighted. The dotted line indicates an FDR of 0.1. *x*-axis, log_2_ fold change of mean gRNA reads per genes comparing treatment to DMSO replicates. *y*-axis, −log_10_ FDR for genes based upon their negative (pink and red dots) and positive (blue dots) fold change and FDR calculated by the MAGeCK algorithm. **C,** Confirmation of deletion of target apoptotic genes following transfection with synthetic crRNA in MSTO-211H, NCI-H28, and H2804 cancer cells. **D,** Six-day viability assay in MSTO-211H cells following deletion of specific apoptotic genes and treatment with either rTRAIL or MEDI3039. *y*-axis: viability effect relative to control cells. **E,** Volcano plots as in **A** and **B** illustrating results of SAM CRISPR activatory screen in PC-9 cells treated with rTRAIL. **F,** MSTO-211H isogenic cell lines were screened versus the parental cell line with a concentration range of 60 compounds and viability measured at day 6. The AUC values for each isogenic cell line and the matched parental Cas9 line were subtracted to calculate a ΔAUC value, with high (positive) values indicating increased resistance to that compound in the isogenic lines, and low (negative) values increased sensitivity. *x*-axis: name of compounds screened. *y*-axis: ΔAUC values.

To determine modes of resistance to TRAIL-induced apoptosis, we conducted a genome-wide CRISPR knockout screen using two TRAIL-sensitive cancer cell lines: the MSTO-211H mesothelioma and PC-9 lung adenocarcinoma models. Cells were transduced with a whole genome human CRISPR/Cas9 library (90,709 sgRNAs targeting a total of 18,010 genes; ref. 9), prior to treatment with rTRAIL. DNA extracted at day 21 for each treatment versus nontreatment pair was analyzed for sgRNAs significantly enriched following drug selection (and therefore targeting putative resistance genes) using the MAGeCK statistical package (Supplementary Tables S1–S4). Gene-level analysis of the sgRNAs most significantly enriched in both the PC-9 and MSTO-211H TRAIL–treated cells were those targeting key nodes of the extrinsic apoptotic signaling pathway, including TRAIL-R2 (*TNFRSF10B*), thereby validating the screen, as well as FADD (*FADD*), BID (*BID*), and caspase-8 (*CASP8*; Fig. 1A and B). Notably, however, sgRNAs targeting the other TRAIL receptor TRAIL-R1 (*TNFRSF10A*) were not enriched, suggesting that TRAIL-R2 is the dominant receptor in both of these models. Gene-set enrichment analysis of KEGG/Reactome pathways identified significant enrichment of apoptosis pathways in both MSTO-211H and PC-9 cells (Supplementary Fig. S1A; Supplementary Table S5). This analysis also identified p53 signaling only in the *TP53* wild-type MSTO-211H cell line, wherein there was significant enrichment of sgRNAs targeting *TP53* and its canonical direct transcriptional target BAX (*BAX*) and in which “intrinsic pathway for apoptosis” was more significantly enriched (Fig. 1A; Supplementary Fig. S1A). This suggests that p53-induced BAX may prime the MSTO-211H cell line for TRAIL-induced apoptosis, which is notable given that caspase-8–activated BID has been reported to preferentially activate BAK rather than BAX (19).

To validate the most significant genes identified across both models as conferring resistance to rTRAIL, we used synthetic crRNA to knock out expression of *FADD*, *BID*, *CASP8*, and *TNFRSF10B* (TRAIL-R2) in the rTRAIL-sensitive MSTO-211H mesothelioma cell line stably expressing Cas9 (Fig. 1C). Deletion of *FADD*, *BID*, *CASP8*, and *TNFRSF10B* not only conferred resistance to rTRAIL, but also resistance to MEDI3039, confirming the results of the CRISPR screen; however, compared with deletion of *FADD*, *CASP8*, and *TNFRSF10B*, deletion of *BID* conferred more partial resistance to MEDI3039 and recombinant forms of TRAIL (Fig. 1C; Supplementary Fig. S1B–S1D). The same effects on MEDI3039 sensitivity were detected in analogous *TNFRSF10B*, *FADD*, *CASP8*, and *BID* cRNA KO models derived from two additional rTRAlL-sensitive mesothelioma cell lines H2804 and NCI-H28 (Supplementary Fig. S1C and S1D).

Given the specificity of the cell death–inducing nature of rTRAIL and the concentration used in the CRISPR knockout screens (Fig. 1A and B), it is perhaps unsurprising that no genes met our significance threshold of FDR < 0.1 for sensitizers in gene-level analysis of the data from the MSTO-211H or PC-9 screens in the CRISPR knockout screens (Fig. 1A and B; Supplementary Tables S6 and S7). However, a parallel genome-wide CRISPR activation screen in PC-9 cells additionally identified negative regulators of cell death MCL-1 and BCL-2L1 (BCL-XL) among the 5 genes below the FDR threshold of 0.1, as potential mediators of resistance to rTRAIL (Fig. 1D). We confirmed the importance of MCL-1 as a potential resistance mechanism using the LoVo colorectal cancer cell line model, which has elevated expression of MCL-1 due to a mutation in *FBXW7*, which encodes an E3 Ligase for MCL-1 (20). In this cell line, which is relatively resistant to MEDI3039, downregulation of MCL-1 increased sensitivity to MEDI3039-induced apoptosis (Supplementary Fig. S1C).

Notably in the CRISPR screens, we did not generally observe significant enrichment in rTRAIL-treated cells for sgRNA targeting genes in other pathways outside of apoptosis (such as ubiquitination, DNA damage repair, cell cycle, or signal transduction), suggesting potential for combining TRAIL receptor–targeted therapies with a broad panel of anticancer agents. To specifically address the potential for cross resistance and to identify potential combination partners to prevent emergence of MEDI3039-resistant cells lacking *CASP8*, *BID*, *FADD*, or *TNFRSF10B*, which we anticipate may emerge clinically, we screened the MSTO-211H, H2804, and NCI-H28 knockout models with a library of 60 compounds (including MEDI3039) targeting diverse cancer pathways, with cell viability assessed at day 6 (Fig. 1E; Supplementary Fig. S1D and S1E; Supplementary Table S8). Resistance to MEDI3039 was confirmed in all the isogenic cell lines, with the BID knockout being the least resistant as noted above; however, importantly, we did not observe cross resistance or indeed any consistent pattern of increased sensitivity to any of the other compounds screened (Fig. 1E; Supplementary Fig. S1C and S1D), suggesting potential for combination with a broad range of anticancer therapies.

### A high-throughput screen of MEDI3039 identifies sensitive subgroups across all tissue lineages and a transcriptional signature of response

**Figure 2.**
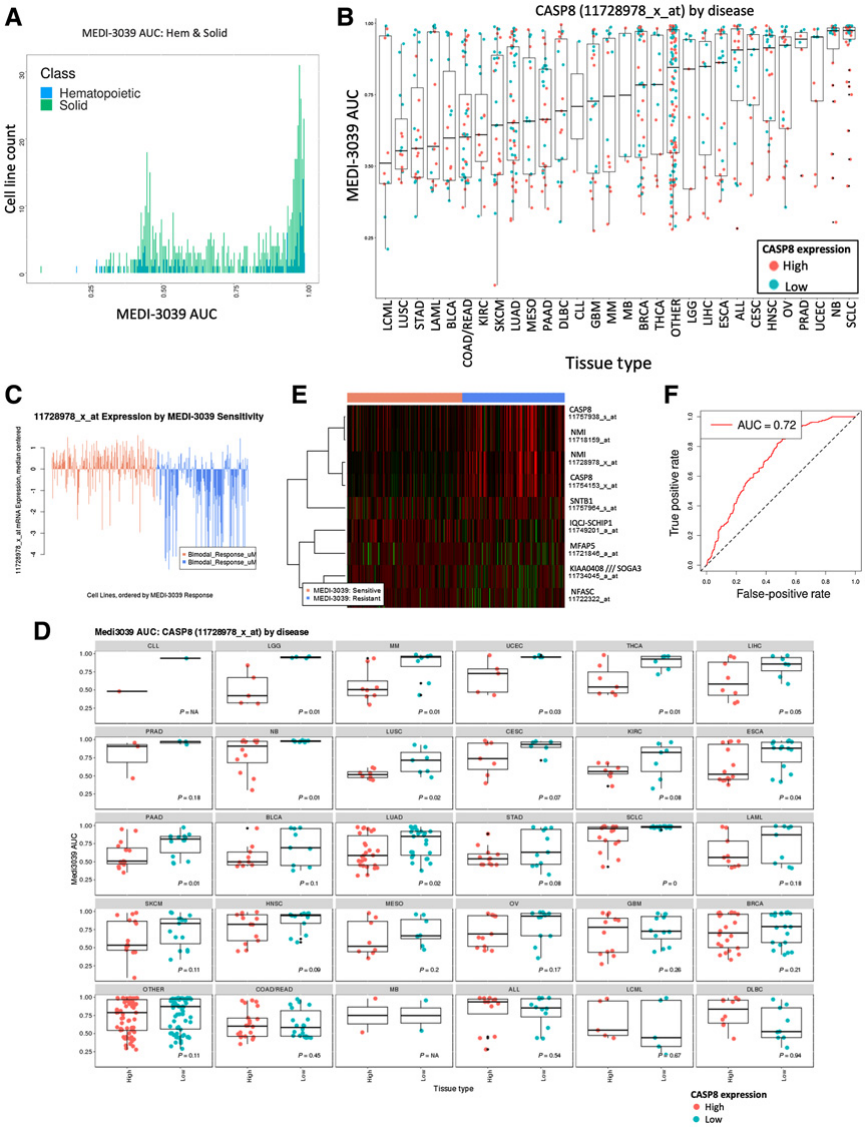
Sensitivity of cancer cell lines to MEDI3039. A panel of 758 cancer cell lines was treated for 6 days with a concentration range of the death receptor agonist MEDI3039 and viability measured as AUC. **A,** Frequency distribution plot illustrating bimodal distribution of MEDI3039 AUC values for solid (green) and hematopoietic (blue) cell lines. **B,** AUC values with SD error bars for each cell line (circles) in 19 tissue types and individually colored according to high or low CASP8 mRNA expression as defined by the bimodal mid-point. **C,** Plot of cell line expression of most significant CASP8 microarray probeset ranked on the basis of sensitivity to MEDI3039. **D,** A two-tailed *t* test was calculated for 30 tissue types using MEDI3039 AUC values and CASP8 expression, and the *P* value indicated (**E**) predictive 9 probeset (7 gene) predictive geneset identified by vSURF random forest analysis of microarray data. **F,** AUC plot demonstrating ability of 9 probeset predictive geneset to classify solid cell lines as “sensitive” or “resistant.” See Supplementary Fig. S2B for description of cancer type abbreviations used in **B** and **D**.

To better understand tissue and genomic determinants underlying sensitivity to multivalent TRAIL-R2 agonists across the spectrum of cancer types, we next assessed response to MEDI3039 across a panel of 758 well-characterized cancer cell lines representing 19 tissue types (Supplementary Fig. S2B; Supplementary Table S9; ref. 9). A consistent feature across most solid and hematologic tumor types was a bimodal pattern of sensitivity, with most cell lines either resistant (high area under the curve, AUC) or sensitive (low AUC) to MEDI3039 (Fig. 2A; Supplementary Fig. S2A). Among the most sensitive tumor types were chronic myeloid leukemia, lung, colorectal, bladder and stomach cancers, with small-cell lung (SCLC) and neuroblastoma the most resistant cancer types (Fig. 2B). To detect statistically significant genomic predictors of sensitivity to MEDI3039, we next carried out ANOVA of MEDI3039 AUCs using as variables the driver mutation status for 270 cancer genes as well as copy-number status (gain or loss) in 470 chromosomal regions recurrently altered in cancer (21). However, we did not identify any genomic alterations that significantly associated with sensitivity or resistance to MEDI3039. We therefore proceeded to test whether transcriptional data for these cell lines could be informative and identify individual transcripts or expression signatures that were significantly associated with MEDI3039 sensitivity. We first defined cell lines within each tissue type as sensitive or resistant using the bimodal mid-point of the density distribution of the AUC values for solid tumor and hematologic cell lines (Fig. 2A; Supplementary Fig. S2A; refs. 11, 12). We did not identify a significant set of genes associated with sensitive hematologic cell lines, whereas comparison of sensitive and resistant solid tumor cell lines identified 642 differentially expressed probesets mapping to 399 genes (fold change >2, FDR < 0.05; Supplementary Table S10). In agreement with the CRISPR rTRAIL screens, gene-set enrichment analysis revealed a strong association of sensitivity with apoptosis, in particular expression of core components of the extrinsic cell death pathway (Supplementary Fig. S2B and S2C; Supplementary Table S11). Most notably, the top 5 probe-sets target caspase-8 (Supplementary Table S12; Fig. 2B–D). TRAIL-R2 (*TNFRS10B*) and Bid (*BID*) were also among the top 25 genes, high expression of which was associated with increased sensitivity to MEDI3039 (Supplementary Table S10). In MEDI3039-sensitive cell lines, there was also significant enrichment of genes expressed in immune signaling, p53 and TNF signaling pathways (Supplementary Fig. S2B; Supplementary Table S11). MEDI3039 resistance was associated with gene expression in neuronal signaling pathways, possibly as a result of the intrinsic resistance observed in the majority of neuroblastoma cell lines (Supplementary Fig. S2B; Supplementary Table S11).

The Random Forest method VSURF (14) was used to further refine the 642 probesets into a minimal set of 9 probesets (mapping to 7 genes) which have the most predictive value for IC_50_ in solid cell lines. Of the 9 predictive probesets, two mapped to *CASP8*, which was also found to be the strongest predictor of response to MEDI3039 (Fig. 2E; Supplementary Table S12). To maximize statistical power, the signature was derived using all solid cell lines across multiple indications. The downside of this approach is that there is clearly a difference in predictive power within different disease settings as exemplified by the performance of the most important variable *CASP8* (Fig. 2B–D). The predictive probeset performs well, classifying cell lines as bimodal low (sensitive) or high (resistant) with an out-of-bag error of 0.28 and an AUC of 0.72 (Fig. 2F). The full drug sensitivity, genomic and transcriptional datasets, and analysis along with predictive gene sets are available to browse and download using a web-based application located at: https://qubapps.shinyapps.io/MEDI3039.

### MEDI3039 sensitivity is significantly affected by FLIP(L)

**Figure 3.**
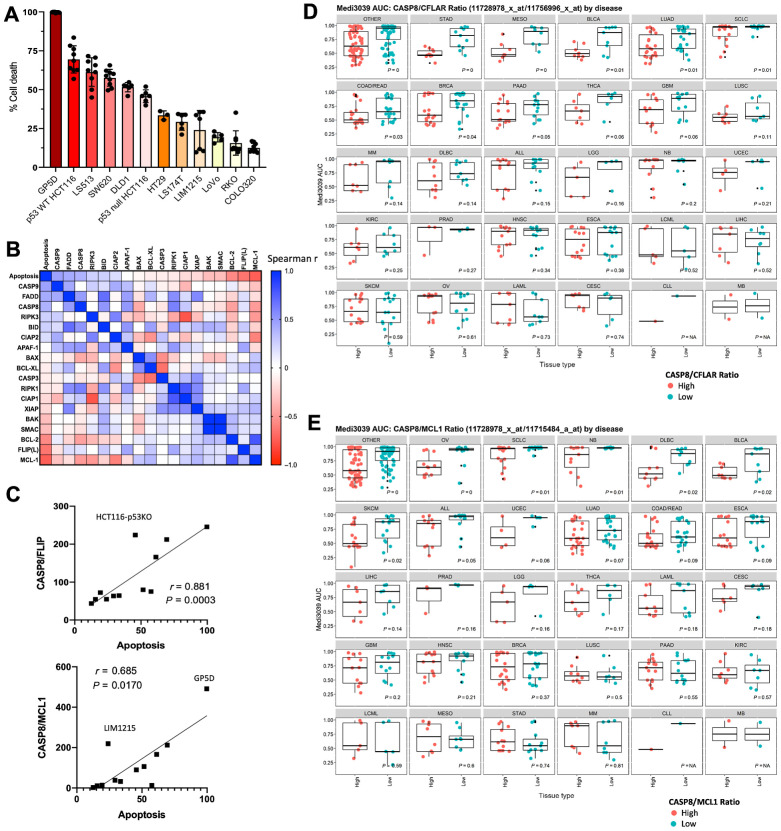
MEDI3039 sensitivity is significantly affected by FLIP(L)–caspase-8 ratio. **A,** Percentage cell death at 24 hours post MEDI3039 10 pmol/L in a panel of colorectal cell lines. **B,** Scatter plot of summary correlation analysis of cell death protein expression and percent cell death induced in response to MEDI3039 across panel of colorectal cancer cell lines. **C,** CASP8/FLIP or CASP8/MCL1 ratio protein expression versus MEDI3039-induced cell death. Each circle indicates a cell line. MEDI3039 AUC for cell lines for different cell types split according to discretized CASP8/FLIP (**D**) or CASP8/MCL1 (**E**) ratio (mRNA expression).

To further determine whether expression of caspase-8 at the protein level or other components of the apoptotic pathway could be used to predict response to MEDI3039, we next used a panel of 12 colorectal cancer cell lines in which we have quantitatively measured protein levels of 18 major cell death regulatory proteins (22). Colorectal cancer cell lines were selected because they exhibited variable sensitivity to MEDI3039 in the cell line screen and interestingly CASP8 mRNA expression alone did not predict for sensitivity to MEDI3039, despite a biomodal pattern of sensitivity (Fig. 2B and D). Similar to viability assays in the cell line screen, the colorectal cancer cell line panel exhibited variable sensitivity to MEDI3039-induced cell death (Fig. 3A), which, in agreement with the data described above, did not correlate with any specific genotype or indeed colorectal cancer transcriptional (CMS/CRIS) transcriptional subtype (23, 24) Consistent with results from the mesothelioma and lung models (Fig. 1), deletion of *CASP8*, *TNFRSF10B* (TRAIL-R2) or *BID* knockout in the MEDI3039-sensitive HCT116 colorectal cancer cell line conferred resistance to MEDI3039 (Supplementary Fig. S3A and S3B), whereas a *FADD* knock out model retained some sensitivity to MEDI3039, potentially because of incomplete loss of FADD in this clone (Supplementary Fig. S3A and S3B). Correlating quantitative analysis of protein levels of core cell death regulators revealed a non-significant correlation between apoptosis induction and pro-caspase-8 expression (Spearman *r* = 0.39, *P* = 0.21; Fig. 3B; Supplementary Fig. S3C), in agreement with the lack of correlation with CASP8 mRNA expression for this disease (Fig. 2E).

FLIP (*CFLAR*, a pseudo-caspase paralogous of and known regulator of caspase-8) is a critical rheostat of cell death induced by the DISCs formed by TRAIL-R2 (25). The short FLIP splice form FLIP(S) is an established inhibitor of TRAIL-R2–induced apoptosis; however, the role of long splice form of FLIP, FLIP(L) is hotly debated, with it being described to act as both an inhibitor and an activator of caspase-8 at the DISC, although our recent study has shed light on this issue (26). Notably, a significant inverse correlation was observed between expression of FLIP(L) and sensitivity to MEDI3039 (Spearman *r* = −0.643; *P* = 0.027; Fig. 3B; Supplementary Fig. S3C); although, no significant association was observed in colorectal cancer between FLIP (*CFLAR*) mRNA expression and sensitivity to MEDI3039 (Supplementary Fig. S3D). However, low expression of FLIP mRNA did significantly correlate with sensitivity to MEDI3039 in ALL, bladder cancer, and diffuse large B-cell lymphoma (DLBCL). Importantly, the ratio between pro-caspase-8 and FLIP(L) was highly correlated with sensitivity to MEDI3039 (Spearman *r* = 0.88, *P* = 0.0003; Fig. 3C), suggesting that in the context of TRAIL-R2 ligation by MEDI3039, FLIP(L) inhibits Caspase-8–mediated apoptosis consistent with our recent findings (26) and reports in other disease types (27, 28). FLIP(S) expression was low or absent in this panel of colorectal cancer cell lines, and its expression did not correlate with response to MEDI3039. This analysis was further supported by the association of higher CASP8/FLIP(L) mRNA ratios with enhanced sensitivity, which was not only significant in colorectal cancer as expected based on the proteomics data, but also in stomach, breast and bladder cancer, mesothelioma, lung adeno- and small-cell carcinoma and pancreatic cancer (Fig. 3D).

No correlations were found between MEDI3039 sensitivity and expression of BID or BAX at the mRNA or protein level (Supplementary Fig. S2A; Supplementary Table S5), despite their knockout inducing resistance to rTRAIL in the CRISPR screens (Fig. 1A and B). However, consistent with our CRISPR Activatory Screen (Fig. 1D), protein expression of antiapoptotic MCL-1 was significantly associated with resistance to MEDI3039 in the colorectal cancer panel (Spearman *r* = −0.73, *P* = 0.008; Supplementary Fig. S3C). However, similar to the observations with FLIP(L), sensitivity to MEDI3039 did not significantly correlate with MCL-1 (*MCL1*) mRNA expression in colorectal cancer, although significant correlations between low MCL-1 mRNA expression and sensitivity to MEDI3039 were observed in DLBCL and bladder cancer (Supplementary Fig. S3E). Moreover, a significant correlation between the CASP8/MCL-1 protein ratio and sensitivity to MEDI3039 was observed in the colorectal cancer panel (Fig. 3C), although this failed to reach significance at the mRNA level (Fig. 3E). However, in several other cancer types, the CASP8/MCL-1 mRNA ratio did significantly correlate with sensitivity to MEDI3039: ovarian cancer, small-cell lung cancer, neuroblastoma, DLBCL, bladder and skin cancers. Notably, these cancer types only partially overlapped with those in which the CASP8/FLIP mRNA ratio was significant (Fig. 3D). Overall, this is consistent with several studies linking MCL-1 to resistance to TRAIL receptor agonists, and our data from high MCL-1–expressing LoVo cells (Supplementary Fig. S1C; refs. 29, 30).

### CASP8 and FLIP expression correlates with response to MEDI3039 in patient-derived xenograft models of colorectal cancer

**Figure 4.**
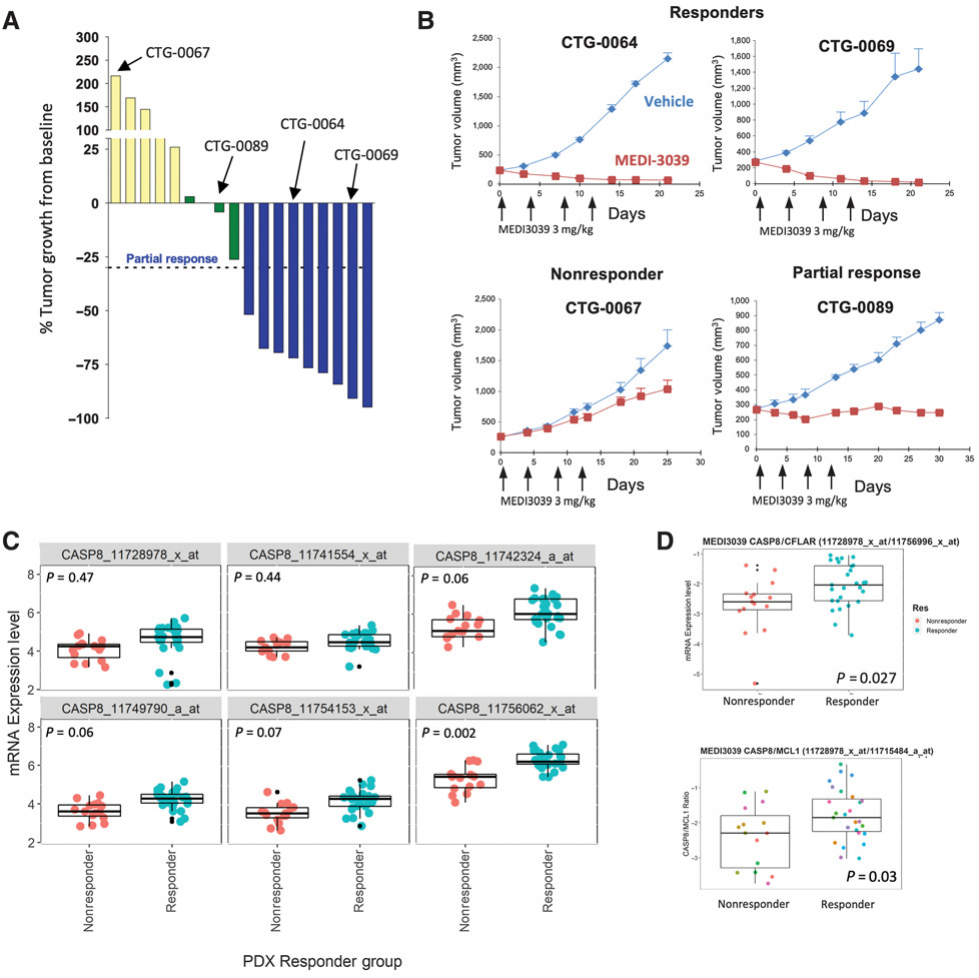
*In vivo* expression of CASP8 and CFLAR (FLIP) modulate response to MEDI3039 in PDX models. **A,** Change in percent tumor growth from baseline was measured at day 18 in 18 CRC PDX models treated with a fixed dose of MEDI3039. **B,** Tumor plots of responder, nonresponder, and partial response models. *x*-axis, days; *y*-axis, tumor volume. Arrows indicate treatment with MEDI3039. Red line, MEDI039-treated mice; blue line, untreated mice. **C,** Tumor CASP8 mRNA expression in PDX nonresponders (red) and responders (blue) with MEDI3039 (each circle represents a tumor from a single mouse). **D,** Comparison of CASP8:CFLAR ratio in MEDI3039 responder (blue) and nonresponder (red) PDX models.

A similar bimodal pattern of sensitivity to MEDI3039 was observed in a panel of 18 colorectal cancer patient-derived xenografts (PDX) treated with single-agent MED3039 (Fig. 4A; Supplementary Table S13). Nine of the 18 models could be classified as Responders based on percentage change tumor volume decrease greater than 30% (*n* = 9 compared with baseline following treatment with MEDI3039), 4 models as partial/minimal response (tumor volume decrease between 5% and 30%, *n* = 4), and 5 models were classified as nonresponders (tumor volume increase >5%, *n* = 5; Fig. 4A and B; Supplementary Fig. S4).

In keeping with the cell line results, no genetic biomarker of sensitivity or resistance was identified, whereas analysis of gene expression data from the PDX models again revealed a significant association between response to MEDI3039 and higher relative levels of CASP8 mRNA (as measured by several array probesets; Fig. 4C; Supplementary Table S14). The significance of this association was increased when the CASP8:FLIP mRNA ratio was used (*P* = 0.027; Fig. 4D), further supporting the potential of this ratio as a predictor of response to second-generation TRAIL-R2 agonists. A similar significant correlation between the CASP8:MCL-1 mRNA ratio and response was observed in these CRC PDX models (Fig. 3E), with a differential pattern of sensitivity likely driven by CASP8 levels.

Overall, these results (Figs. 1–3) indicate that, although there are no genetic biomarkers to predict response to multivalent TRAIL-R2 agonists, mRNA and, in particular, protein expression levels of caspase-8 relative to FLIP and/or MCL-1 may be useful patient stratifying predictive biomarkers in at least a subset of solid tumors.

### Identification of synergistic combination partners for MEDI3039

**Figure 5.**
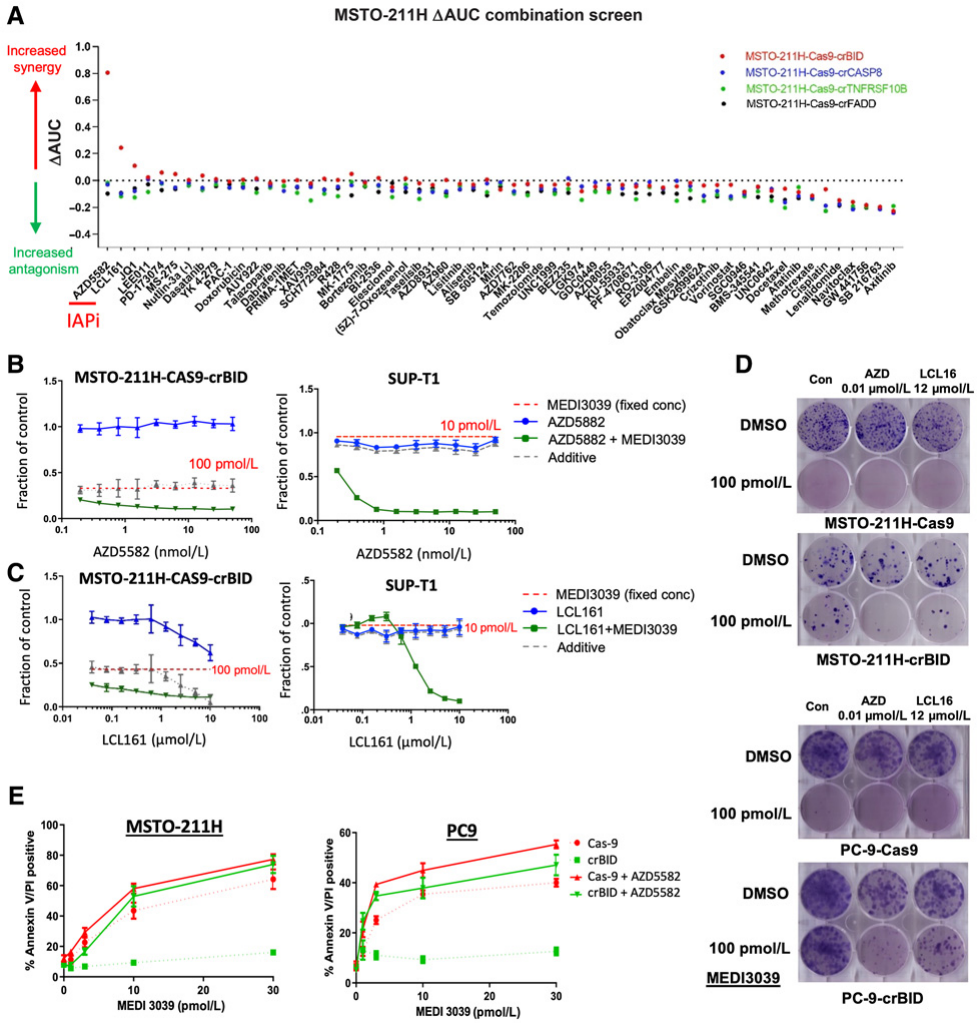
Drug combinations to overcome rTRAIL resistance in isogenic cell lines. **A,** Isogenic MSTO-211H cell lines were screened against 59 compounds in combination with a fixed dose of MEDI3039 (100 pmol/L). Viability was measured at day 6. For each combination, a ΔAUC was calculated by subtracting the observed from the expected AUC (based upon the activity of the MEDI3039 concentration as a single agent). Values >0.2 are indicative of synergy. *x*-axis: name of compounds screened. Bid-KO MSTO-211H or Bid-mutant Sup-T1 cells were treated with a concentration range of the IAP inhibitor AZD5582 (**B**) or LCL161 (**C**) for 6 days (blue line) or in combination with a fixed concentration of MEDI3039 (green, IC_90_ values of parental cell line). Indicated is the effect of the fixed concentration of MEDI3039 (red dotted) and the expected (additive) effect of the combination (gray dotted). *x*-axis: log_10_ scale concentration range. *y*-axis: relative viability effect. **D,** Clonogenic survival assays at day 14 in Cas9 versus crBID MSTO-211H and PC-9 cells treated with the indicated IAP inhibitors AZD and LCL161 as single agents or combined with MEDI3039. AZD, AZD5582. **E,** Annexin V/PI staining of Cas9 versus BID KO MSTO-211H and PC-9 cells following 24-hour treatment with MEDI3039 ±10 nmol/L AZD5582.

Finally, in order to identify potential strategies that could reverse likely mechanisms of clinical resistance, we screened 60 compounds with varying mechanisms of action (including a number of cell death targeting agents) in combination with a fixed concentration of MEDI3039 in MSTO-211H *FADD*, *BID*, *CASP8*, and *TNFRSF10B* deleted MSTO-211H, NCI-H28, and H2804 cells (Fig. 1C). For each combination, a “ΔAUC” was calculated by subtracting the observed from the expected AUC (based on the activity of the MEDI3039 concentration as a single agent). Synergy was observed with 2 IAP inhibitors (LCL161 and AZD5582), but only in the BID-deleted models (Fig. 5A; Supplementary Fig. S5A and S5B; Supplementary Tables S14 and S15). Repeating these analyses in 2 further models, NCI-H28 and NCI-2804, we obtained similar results, with LCL161 and AZD5582 reproducibly synergizing with MEDI3039, but only in the BID-deleted models screened (Supplementary Fig. S5A and S5B); this was further confirmed in a *BID*-deleted PC9 lung cancer cell line (Supplementary Fig. S5C and S5D). Moreover, in the panel of 758 cell lines screened with MEDI3039, we detected two cell lines (SUP-T1 and MFE-319) harboring constitutive truncating (loss-of-function) mutations in BID (p.E98* and p.G16fs*22, respectively), and both were resistant to MEDI3039 and rTRAIL (Supplementary Table S9; Supplementary Fig. S5E).

Importantly, we observed strong synergy when combining MEDI3039 with either LCL161 or AZD5582 across all BID-deleted/mutated models examined (Fig. 5B and C; Supplementary Fig. S5C and S5D) which correlated with significant reduction in long-term survival (Supplementary Fig. S6A). Cell death–inducing effects of IAP inhibitor/MEDI3039 combinations was confirmed in MSTO-211H, NCI-H28, NCI-2804, and PC-9 BID–deleted models (Fig. 5E; Supplementary Fig. S6B). In all models, we observed that the total or partial resistance to MEDI3039 conferred by loss of BID (which notably was cleaved in each parental model by MEDI3039), was either partially or totally overcome by cotreatment with an IAP antagonist.

## Discussion

Apoptotic cell death is a key barrier to cancer development; therefore, evasion of apoptosis is a key hallmark of many cancers (31). Early attempts to target proapoptotic effectors involved the use of ligands to activate the death receptor family of cell surface proteins, in particular TRAIL-R1 and TRAIL-R2 (1). Recognition that apoptosis induction is controlled by dynamic interactions between pro- and antiapoptotic members of the Bcl-2 family has led to active research programs that target antiapoptotic members of this family; the first of these, venetoclax, has been approved for treatment of chronic lymphocytic leukemia (CLL) with 17p deletion (32). Several inhibitors of the IAPs have also been developed, with the second generation of these agents now in early-phase clinical trials (33–36).

Two challenges lie in wait for any novel therapeutic in the clinic; how to identify the patient subpopulations likely to show greatest response to the drug and how to avoid/overcome likely emergence of drug resistance in those patients who initially respond to treatment. In this study, we identified a transcriptional signature strongly associated with response to a TRAIL-R2 agonist that is dominated by genes involved in apoptosis. As the cell death pathway is regulated both positively and negatively by several genes, we reasoned that a multi-gene or multi-protein signature would likely be more predictive than expression of any single gene in this pathway. Indeed, the ratio of caspase-8 to FLIP, or caspase-8 to MCL-1 were more predictive of response to MEDI3039 in several major cancer types compared with single biomarker analyses when using either protein or mRNA expression levels. The current batch of clinical studies of TRAIL-R2 agonists in patients with cancer could be used to assess whether either of these ratios, or an algorithm taking into account all three biomarkers is indeed predictive of response to treatment. It is also pertinent to note that for some cancer therapeutics, such as TRAIL-R agonists where there is no genetic link to response, predictive biomarkers may only be measured in tissue samples from the tumor itself, and indirect measurements (such as circulating free DNA) are not informative.

The emergence of drug resistance is an almost universal phenomenon in the cancer treatment paradigm. Understanding which genes are complicit in the development of resistance allows the possibility of adopting therapeutic strategies to either prevent resistance or treat resistant cells. In our study, we demonstrated that deletion of direct effectors of the TRAIL-R2 pathway (the receptor itself, FADD, caspase-8, and BID) conferred resistance. One of these potential mechanisms of resistance, loss of BID expression, could be overcome, with the addition of an IAP inhibitor resensitizing to the TRAIL-R2 agonist. In these BID-deficient models, the IAP inhibitor may work by inhibiting XIAP and converting these cells into “Type-I”-like models in which caspase-8 can directly induce apoptosis without involvement of BID-mediated activation of the intrinsic mitochondrial pathway. Thus, IAP inhibitors may be clinically useful combination partners for second-generation TRAIL-R2 agonists, a finding in agreement with a recent study in melanoma (37).

In cell lines in which we modeled the emergence of resistance to second-generation multivalent TRAIL-R2 agonists by deleting caspase-8, FADD, BID, or TRAIL-R2 itself, we did not observe cross-resistance or indeed any consistent pattern of increased sensitivity to any of 60 other anticancer compounds screened. Taken together with results from the CRISPR screens, these results demonstrate that there is a limited repertoire of potential resistance mechanisms to second-generation TRAIL-R2 agonists and, most importantly from a clinical standpoint, these resistance mechanisms do not appear to overlap with those of many other standard anticancer agents. However, recent studies indicate that CAR-T cell–mediated elimination of B-cell malignancies is FADD- and TRAIL-R2 dependent; therefore, there is potential for cross resistance with this type of therapy (38).

Notably, sgRNAs targeting *CFLAR*/FLIP or *MCL1*/MCL-1 were not significantly depleted in the CRISPR KO screens (Fig. 1A and B; Supplementary Tables S1–S5); this is likely because these antiapoptotic genes are frequently essential for long-term survival even in the absence of TRAIL treatment, therefore there may be little differential selection pressure in the DMSO- and rTRAIL-treated groups (39, 40). Indeed, our previous findings using siRNA (41) and analysis of DepMap CRISPR data (https://depmap.org/portal/; ref. 42) indicate that *CFLAR* CRISPR deletion is “strongly selective” or “essential” in more than half the 700+ cell lines screened. In fact, the MSTO-211H model used in the rTRAIL CRISPR screen is highly FLIP dependent (there are no DepMap data for PC-9 cells). The MSTO-211H model is also MCL-1 dependent. Both FLIP and MCL-1 are highly turned over at the protein level (7, 43) which may explain why their protein levels but not their mRNA levels correlated more strongly with sensitivity to MEDI3039 in the colorectal cancer panels.

In summary, we provide the first comprehensive functional genomics analysis of the efficacy of second-generation TRAIL-R2 agonists. While sensitivity to these agents does not correlate with any genomic alterations, the most sensitive solid tumor cells had a high level of expression of the key effector caspase of the pathway, caspase-8, relative to direct (FLIP) and indirect (MCL-1) pathway inhibitors. Notably however, no transcriptomics-based predictive biomarkers for use of second-generation TRAIL-R2 agonists in hematologic malignancies was revealed by our studies. Furthermore, our global CRISPR screens suggest that loss of key effectors like BID, FADD, and caspase-8 will likely drive clinical resistance to second-generation TRAIL-R2 agonists. In this scenario, strategies to reestablish expression of these effectors (for example with epigenetic modifying agents), or targeting the vulnerabilities induced by their loss may be effective; for example, caspase-8–deficient cells may become hypersensitive to necroptosis, a form of cell death that can be triggered by IAP inhibitors that is normally inhibited by caspase-8 (44). Moreover, the lack of cross-resistance to other anticancer agents in MEDI3039-resistant models suggests that there are a range of potential combination partners for second-generation TRAIL-R2 agonists with orthogonal mechanisms of action.

## Authors' Disclosures

A. Wesa reports other support from Champions Oncology during the conduct of the study; and other support from Champions Oncology outside the submitted work. D.A. Tice reports a patent for WO2011130328A1 issued. L.F. Wessels reports grants from Genmab BV outside the submitted work. M. Garnett reports grants from Wellcome during the conduct of the study. U. McDermott reports other support from AstraZeneca during the conduct of the study; and other support from AstraZeneca outside the submitted work. No disclosures were reported by the other authors.

## Supplementary Material

Supplementary Figure

Supplementary Data
