# Supplementary material for: Functional Genomic Identification of Predictors of Sensitivity and Mechanisms of Resistance to Multivalent Second-Generation TRAIL-R2 Agonists
Source: Mol Cancer Ther. 2022 Jan 27;21(4):594–606. doi: 10.1158/1535-7163.MCT-21-0532 (PMC7612587; doi:10.1158/1535-7163.MCT-21-0532)
Supplement: Supplementary Figure [file mct-21-0532_supplementary_figures_supp0.pdf]

A

	TOP 5 Pathways MSTO-211H	SIZE	NOM p-val	FDR q-val	FWER p-val
KEGG	P53_SIGNALING_PATHWAY	67	0.000	0.068	0.067
	APOPTOSIS	85	0.000	0.066	0.123
	BIOSYNTHESIS_OF_UNSATURATED_FATTY_ACIDS	22	0.005	0.127	0.311
	AMYOTROPHIC_LATERAL_SCLEROSIS_ALS	51	0.000	0.303	0.717
	ABC_TRANSPORTERS	44	0.005	0.280	0.762
Reactome	INTRINSIC_PATHWAY_FOR_APOPTOSIS	29	0.000	0.015	0.014
	ACTIVATION_OF_BH3_ONLY_PROTEINS	16	0.002	0.033	0.065
	DESTABILIZATION_OF_MRNA_BY_BRF1	16	0.003	0.189	0.429
	SIGNALING_BY_NODAL	17	0.011	0.506	0.870
	DESTABILIZATION_OF_MRNA_BY_TRISTETRAPROLIN_TTP	16	0.013	0.554	0.940

	TOP 5 Pathways PC-9	SIZE	NOM p-val	FDR q-val	FWER p-val
KEGG	APOPTOSIS	85	0.000	0.318	0.260
	RIG_I_LIKE_RECEPTOR_SIGNALING_PATHWAY	71	0.000	0.183	0.296
	PROTEIN_EXPORT	23	0.019	0.321	0.605
	MATURITY_ONSET_DIABETES_OF_THE_YOUNG	25	0.025	0.430	0.806
	P53_SIGNALING_PATHWAY	67	0.004	0.395	0.849
Reactome	CTLA4_INHIBITORY_SIGNALING	21	0.000	0.140	0.128
	ADP_SIGNALLING_THROUGH_P2RY12	21	0.003	0.165	0.283
	G_BETA_GAMMA_SIGNALLING_THROUGH_PI3KGAMMA	24	0.005	0.206	0.463
	MAPK_TARGETS_NUCLEAR_EVENTS_MEDIATED_BY_MAP_KINASES	30	0.001	0.196	0.541
	INTRINSIC_PATHWAY_FOR_APOPTOSIS	29	0.002	0.174	0.578

B

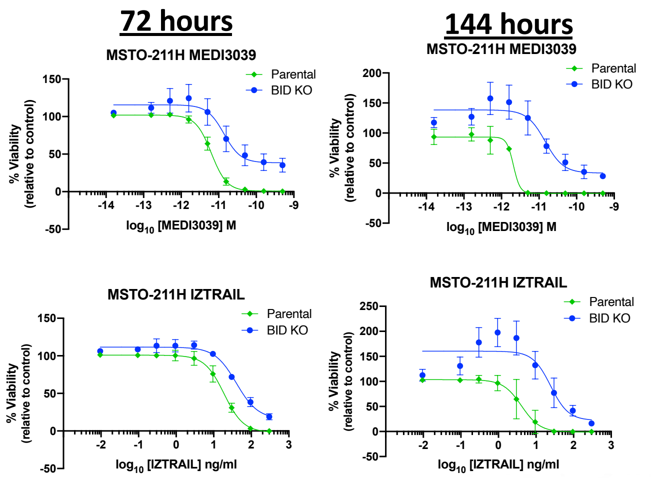

C

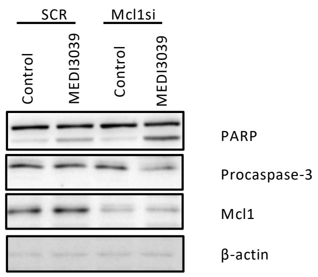

D

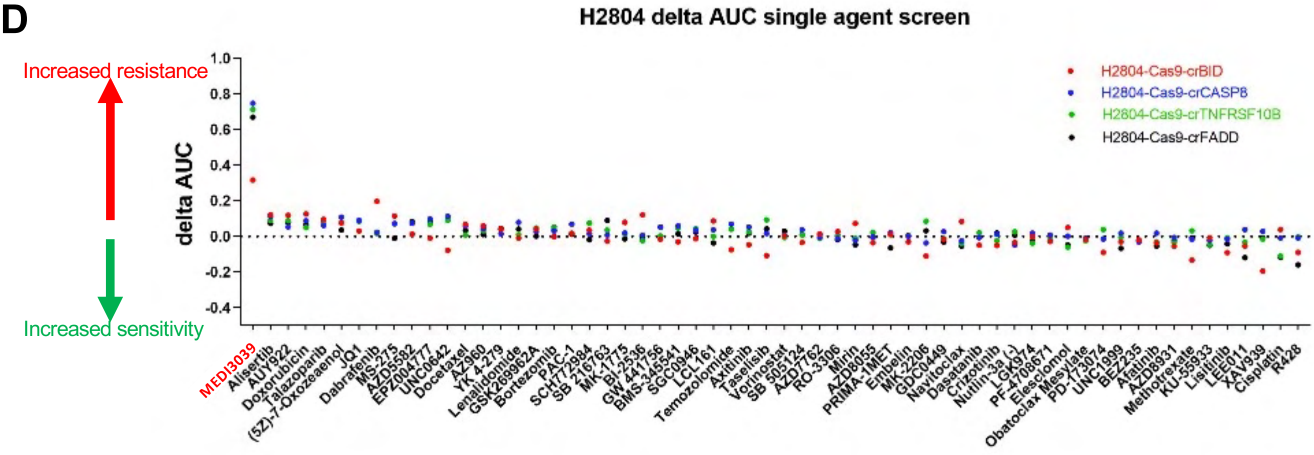

E

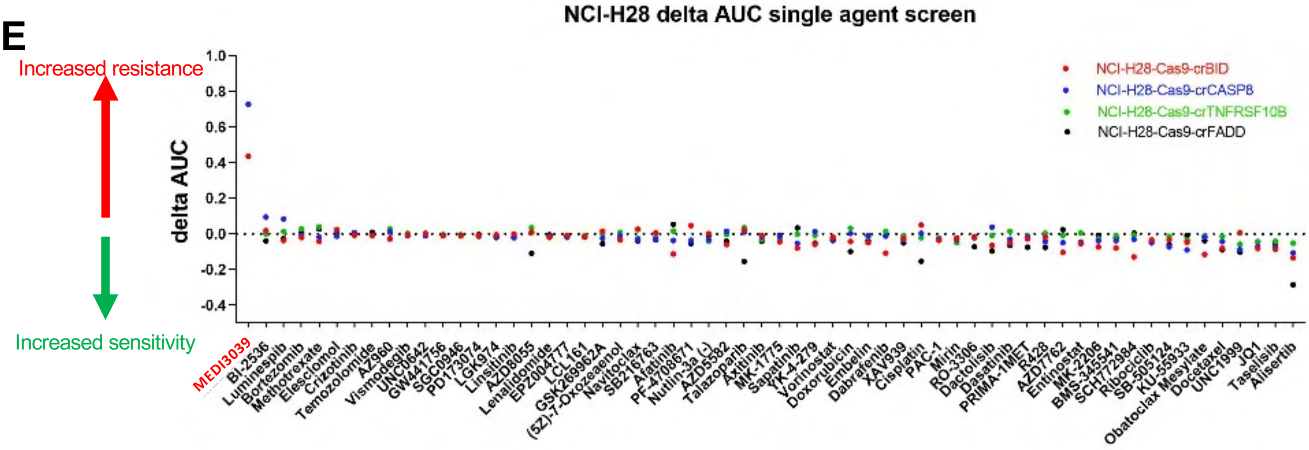

Figure S1 | Silencing of apoptotic pathway genes confers resistance to agonists of the death receptor pathway. (**A**) GSEA analysis of top enriched TRAIL resistance genes for MSTO-211 and PC-9 cell lines. (**B**) Dose response curves comparing effects of MEDI3039 and izTRAIL in MSTo-211H cell lines at 72 and 144 h. (C) Western blot analysis of of effects of MCL1 depletion on parp cleavage in HCT116 cells treated with 1nM MEDI3039 (**D/E**) Each of the H2804 (**C**) and NCI-H28 (D) isogenic cell lines was screened versus the parental cell line with a concentration range of 60 compounds and viability measured at day 6. The AUC (area-under-the-curve) values for each isogenic cell line and the matched parental Cas9 line were subtracted to calculate a deltaAUC value, with high (positive) values indicating increased resistance to that compound in the isogenic lines, and low (negative) values increased sensitivity. X-axis - name of compounds screened. Y-Axis - delta AUC values.

A

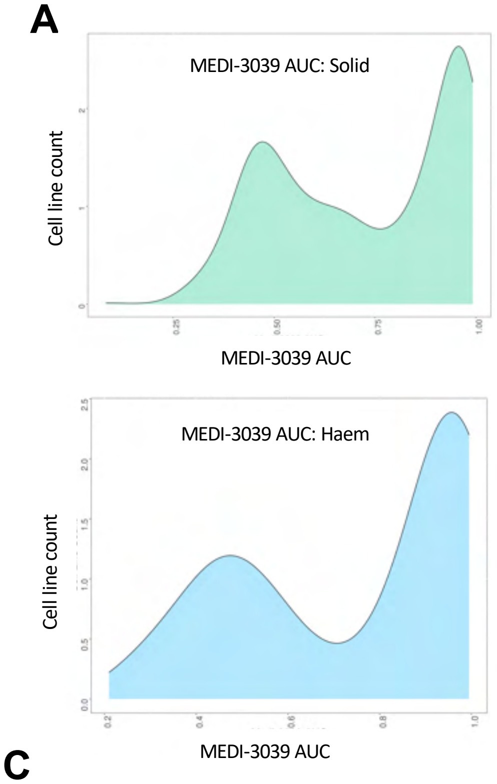

B

Abbreviation	TCGA Cancer Type
LAML	Acute Myeloid Leukemia
ACC	Adrenocortical carcinoma
BLCA	Bladder Urothelial Carcinoma
LGG	Brain Lower Grade Glioma
BRCA	Breast invasive carcinoma
CESC	Cervical squamous cell carcinoma and endocervical adenocarcinoma
CHOL	Cholangiocarcinoma
LCML	Chronic Myelogenous Leukemia
COAD	Colon adenocarcinoma
CNTL	Controls
ESCA	Esophageal carcinoma
GBM	Glioblastoma multiforme
HNSC	Head and Neck squamous cell carcinoma
KICH	Kidney Chromophobe
KIRC	Kidney renal clear cell carcinoma
KIRP	Kidney renal papillary cell carcinoma
LIHC	Liver hepatocellular carcinoma
LUAD	Lung adenocarcinoma

Abbreviation	TCGA Cancer Type
LUSC	Lung squamous cell carcinoma
DLBC	Lymphoid Neoplasm Diffuse Large B-cell Lymphoma
MESO	Mesothelioma
MISC	Miscellaneous
OV	Ovarian serous cystadenocarcinoma
PAAD	Pancreatic adenocarcinoma
PCPG	Pheochromocytoma and Paraganglioma
PRAD	Prostate adenocarcinoma
READ	Rectum adenocarcinoma
SARC	Sarcoma
SKCM	Skin Cutaneous Melanoma
STAD	Stomach adenocarcinoma
TGCT	Testicular Germ Cell Tumors
THYM	Thymoma
THCA	Thyroid carcinoma
UCS	Uterine Carcinosarcoma
UCEC	Uterine Corpus Endometrial Carcinoma
UVM	Uveal Melanoma

C

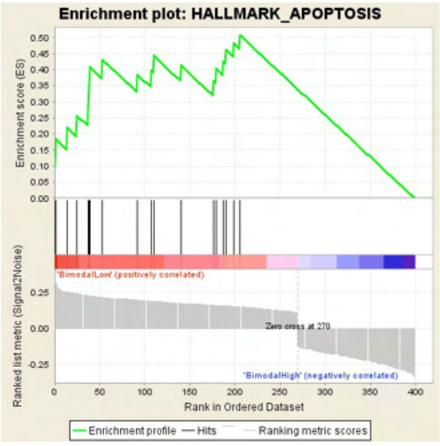

D

Gene sets associated with sensitivity to MEDI3039	Category	SIZE	ES	NES	NOM p-val	FDR q-val
HALLMARK_INFLAMMATORY_RESPONSE	Bimodal_Low	16	0.5076	1.1994	0.2652	1
HALLMARK_APOPTOSIS	Bimodal_Low	18	0.5052	1.1414	0.3909	1
HALLMARK_INTERFERON_ALPHA_RESPONSE	Bimodal_Low	28	0.4754	1.0665	0.4847	1
HALLMARK_TNFA_SIGNALING_VIA_NFKB	Bimodal_Low	27	0.4154	1.0457	0.4519	1
REACTOME_INNATE_IMMUNE_SYSTEM	Bimodal_Low	15	0.4425	1.0366	0.4612	1
HALLMARK_P53_PATHWAY	Bimodal_Low	14	0.4200	1.0245	0.4799	1
REACTOME_INTERFERON_GAMMA_SIGNALING	Bimodal_Low	11	0.4523	1.0225	0.4743	1
HALLMARK_INTERFERON_GAMMA_RESPONSE	Bimodal_Low	38	0.4193	1.0142	0.5041	1
REACTOME_INTERFERON_ALPHA_BETA_SIGNALING	Bimodal_Low	17	0.4458	0.9477	0.5772	1
REACTOME_INTERFERON_SIGNALING	Bimodal_Low	22	0.4129	0.9465	0.5444	1
REACTOME_CYTOKINE_SIGNALING_IN_IMMUNE_SYSTEM	Bimodal_Low	25	0.4006	0.9356	0.5676	1
**Gene sets associated with resistance to MEDI3039**	**Category**	**SIZE**	**ES**	**NES**	**NOM p-val**	**FDR q-val**
REACTOME_AXON_GUIDANCE	Bimodal_High	13	-0.4662	-1.1853	0.2606	1.0000
REACTOME_NEURONAL_SYSTEM	Bimodal_High	11	-0.5343	-1.1812	0.3093	0.8407
REACTOME_DEVELOPMENTAL_BIOLOGY	Bimodal_High	19	-0.4063	-1.1710	0.2729	0.5789
KEGG_CELL_ADHESION_MOLECULES_CAMS	Bimodal_High	13	-0.2300	-0.6280	0.9032	1.0000
HALLMARK_ESTROGEN_RESPONSE_LATE	Bimodal_High	14	-0.2185	-0.6141	0.9284	1.0000
HALLMARK_KRAS_SIGNALING_UP	Bimodal_High	29	-0.1528	-0.4877	0.9941	1.0000
HALLMARK_APICAL_JUNCTION	Bimodal_High	16	-0.1318	-0.3958	1.0000	0.9919

Figure S2 | Sensitivity of cancer cell lines to MEDI3039. (A) Bimodal distribution of IC50 values for MEDI3039 in solid (Green) versus haematological (Blue) cancers. (B) Table describing cancer types abbreviations used in figure **(C/D)** Gene set enrichment analysis for genes differentially expressed in sensitive versus resistant cell lines.

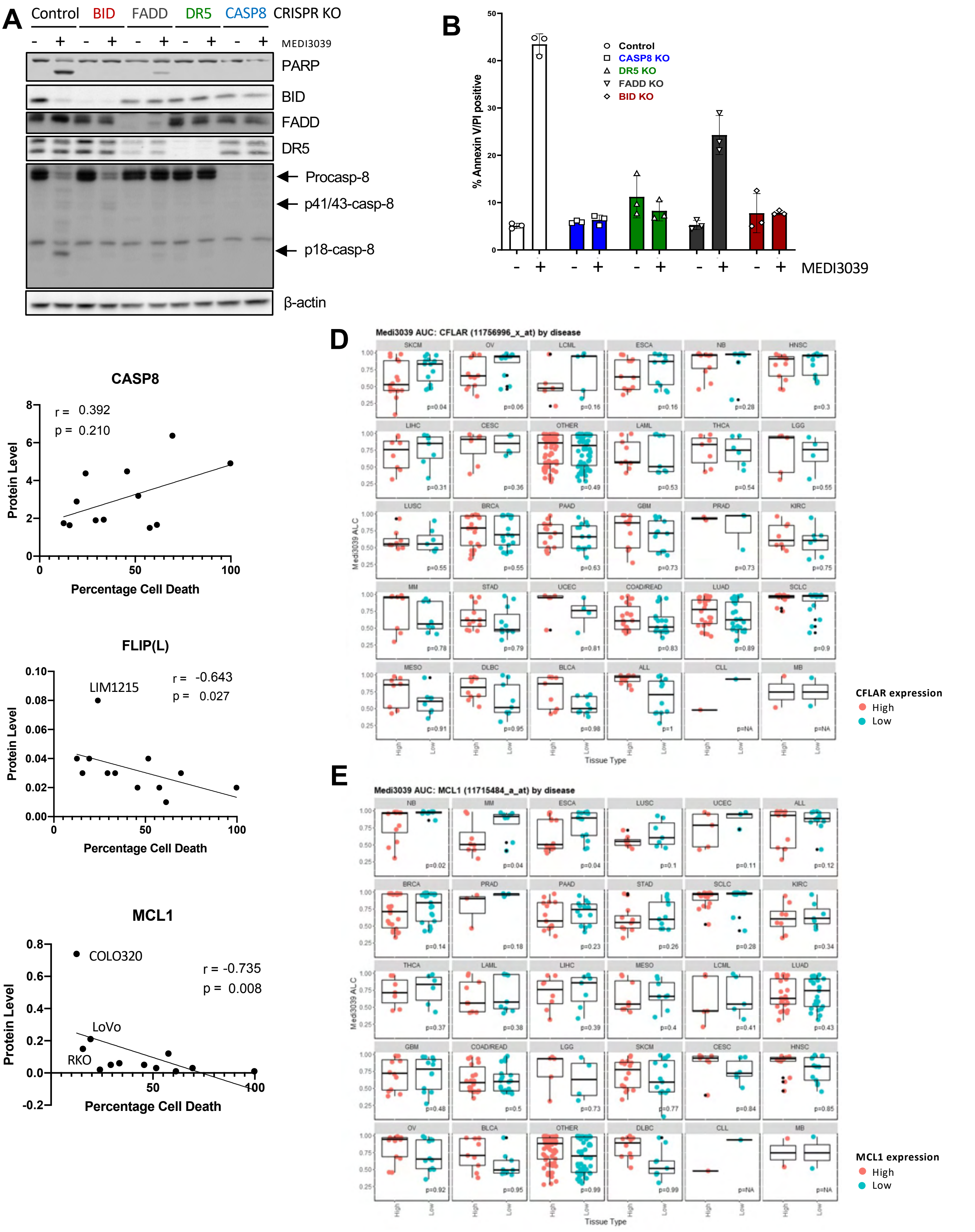

Figure S3 | MEDI3039 sensitivity is significantly affected by FLIP(L). Western blot (A) and high content microscopy (B) analyses of cell death induced by 100pM MEDI3039 treatment for 24h in control and CRISPR knockout HCT116 colorectal cancer cell lines. (C) CASP8/FLIP or CASP8/MCL1 ratio protein expression versus MEDI3039-induced cell death. (D) MEDI3039 AUC for cell lines for different cell types split according to discretised CASP8 or FLIP mRNA expression

Supplementary Figure 4

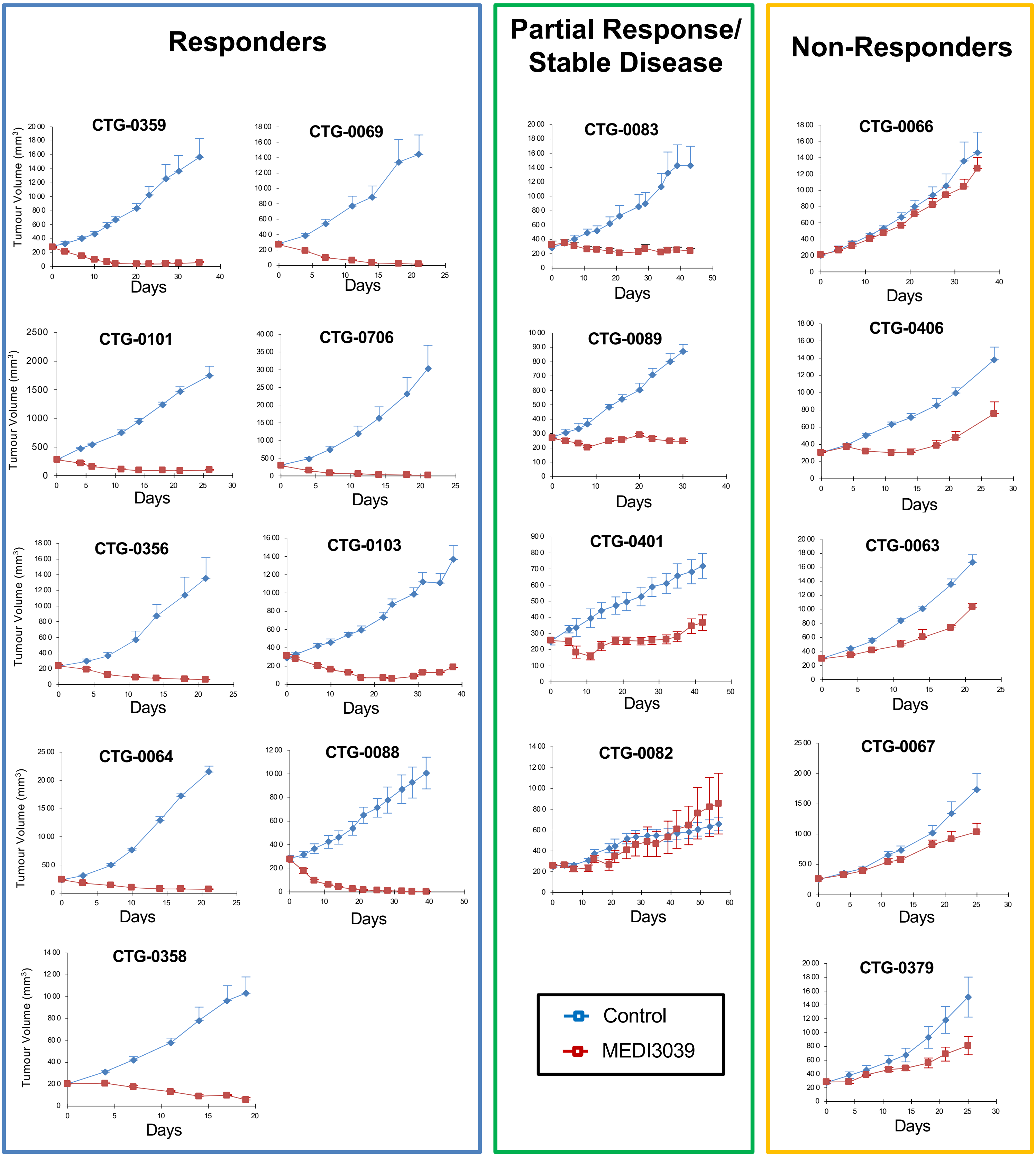

Figure S4 | MEDI3039 treatment effect in a panel of CRC PDX models. Tumour volume measurements measured in 18 CRC PDX models treated with 3mg/kg intraperitoneally with MEDI3039 twice per week for 2 weeks. X-axis – days. Y-axis – tumour volume (mm^3) . Error bars = SEM.

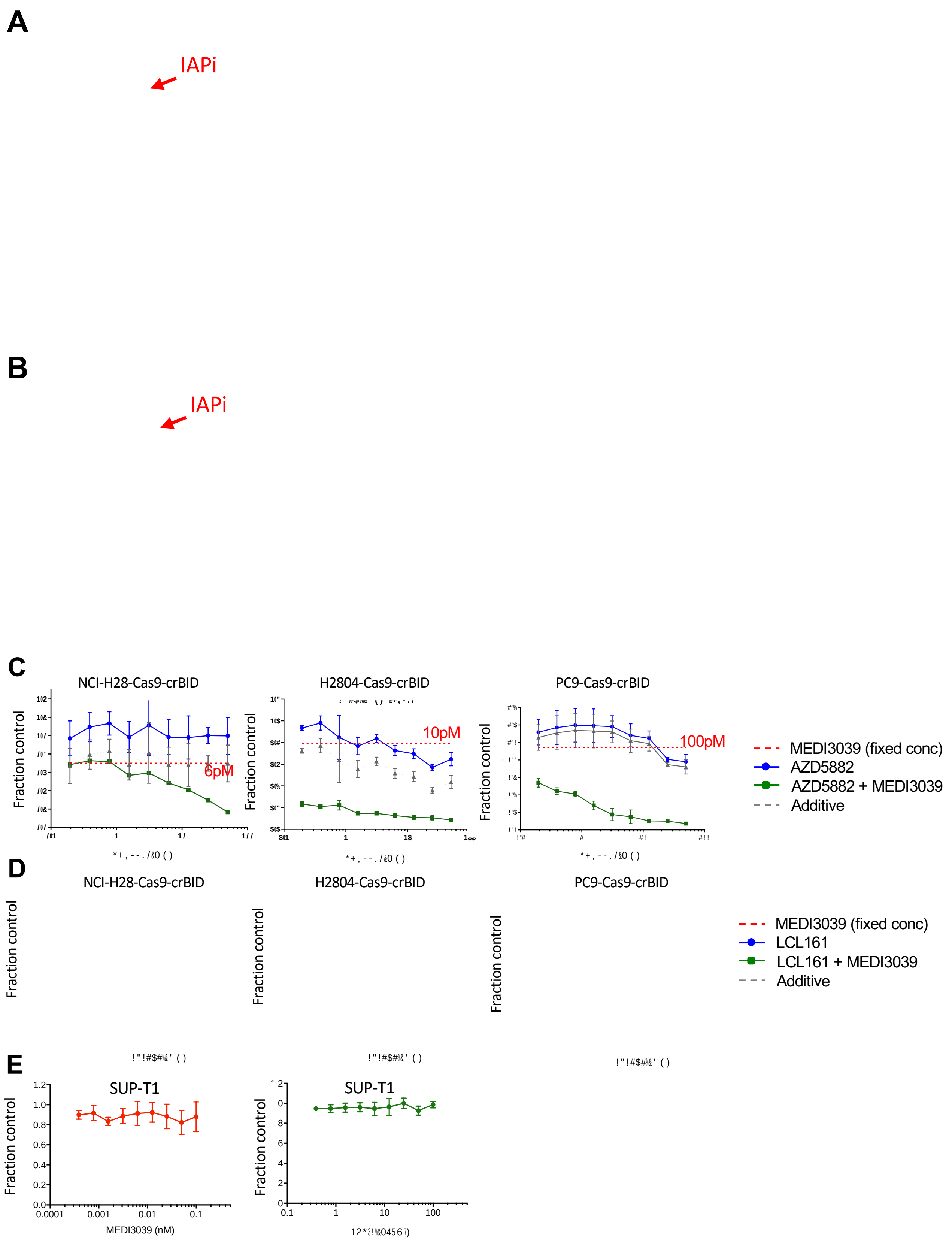

Figure S5 | IAP inhibition re-sensitizes to MEDI3039 in BID-deleted lung cancer cells. (A/B) Isogenic NCI-H28 or H2804 cell lines were screened against 59 compounds in combination with a fixed dose of MEDI3039 (100pM). Viability was measured at day 6. For each combination, a deltaAUC was calculated by subtracting the observed from the expected AUC (based upon the activity of the MEDI3039 concentration as a single agent). Values >0.2 are indicative of synergy. X-axis - name of compounds screened. Bid-KO NCI-H28, H2804 or PC-9 cells were treated with a concentration range of the IAP inhibitor AZD5582 (C) of LCL161 (D) for 6 days (blue line) or in combination with a fixed concentration of MEDI3039 (green, IC90 values of parental cell line). Indicated is the effect of the fixed conc. of MEDI3039 (red dotted) and the expected (additive) effect of the combination (grey dotted). X-axis - log10 scale concentration range. Y-axis - relative viability effect. (E) Bid mutant SUP-T1 cells were treated with a concentration range of MEDI3039 or rTRAIL.

A

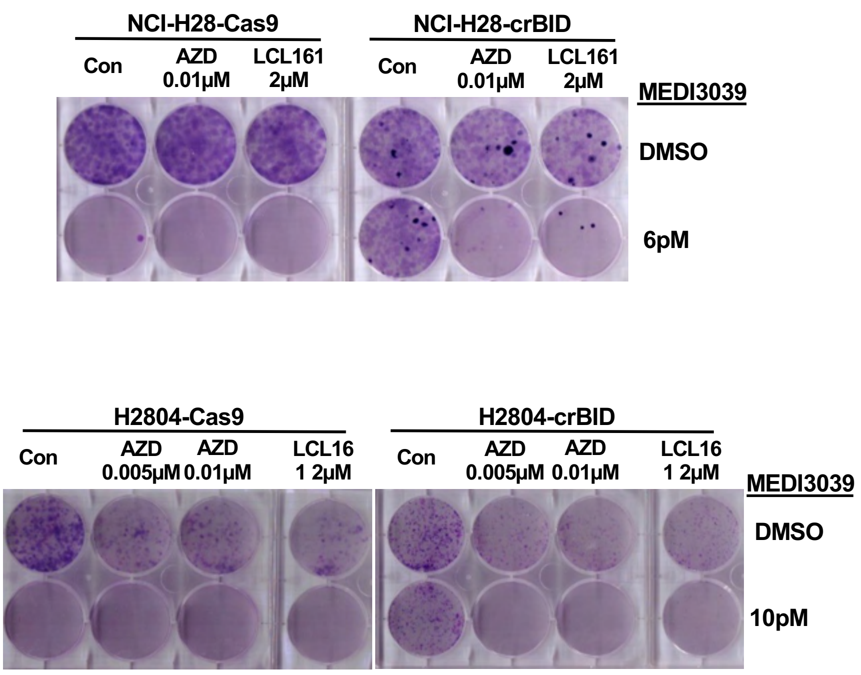

B

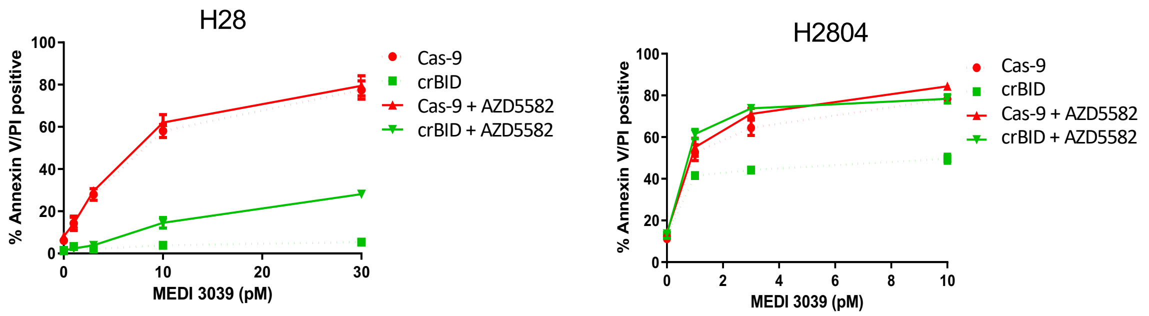

Figure S6 | IAP inhibition re-sensitizes to MEDI3039 in BID-deleted lung cancer cells. (A) Clonogenic survival assays at day 14 in Cas9 vs crBID NCI-H28 AND H2804 cells treated with the indicated IAP inhibitors AZD and LCL161 as single agents or combined with MEDI3039 (IC90 concentration). Abbreviations - AZD, AZD5582. (B) AnnexinV/PI staining of Cas9 vs BID KO NCI-H28 AND H2804 cells following 24h treatment with MEDI3039 +/-10nM AZD5582
